# Joint analysis of histopathology image features and gene expression in breast cancer

**DOI:** 10.1186/s12859-016-1072-z

**Published:** 2016-05-11

**Authors:** Vlad Popovici, Eva Budinská, Lenka Čápková, Daniel Schwarz, Ladislav Dušek, Josef Feit, Rolf Jaggi

**Affiliations:** Institute of Biostatistics and Analyses, Faculty of Medicine, Masarykova Univerzita, Kamenice 5, Brno, 62500 Czech Republic; RECETOX, Masarykova Univerzita, Kamenice 5, Brno, 62500 Czech Republic; Department of Clinical Research, Faculty of Medicine, University of Bern, Bern, Switzerland

**Keywords:** Histopathology images, Image analysis, Biomarker discovery, Gene expression, Multimodal data mining

## Abstract

**Background:**

Genomics and proteomics are nowadays the dominant techniques for novel biomarker discovery. However, histopathology images contain a wealth of information related to the tumor histology, morphology and tumor-host interactions that is not accessible through these techniques. Thus, integrating the histopathology images in the biomarker discovery workflow could potentially lead to the identification of new image-based biomarkers and the refinement or even replacement of the existing genomic and proteomic signatures. However, extracting meaningful and robust image features to be mined jointly with genomic (and clinical, etc.) data represents a real challenge due to the complexity of the images.

**Results:**

We developed a framework for integrating the histopathology images in the biomarker discovery workflow based on the bag-of-features approach – a method that has the advantage of being assumption-free and data-driven. The images were reduced to a set of salient patterns and additional measurements of their spatial distribution, with the resulting features being directly used in a standard biomarker discovery application. We demonstrated this framework in a search for prognostic biomarkers in breast cancer which resulted in the identification of several prognostic image features and a promising multimodal (imaging and genomic) prognostic signature. The source code for the image analysis procedures is freely available.

**Conclusions:**

The framework proposed allows for a joint analysis of images and gene expression data. Its application to a set of breast cancer cases resulted in image-based and combined (image and genomic) prognostic scores for relapse-free survival.

**Electronic supplementary material:**

The online version of this article (doi:10.1186/s12859-016-1072-z) contains supplementary material, which is available to authorized users.

## Background

The recent technological progress made scanning the whole pathology slides affordable and its integration in the routine pathology workflow feasible. This resulted in a revived interest in developing new computational methods for nuclear morphometry and tissue architecture characterization, as well as for developing new tissue-based biomarkers [1]. In the last decade, genomic and proteomic techniques have been the methods of choice for novel biomarker discovery. When applied to the same sample, the pathology imaging and *omics technologies allow the investigation of the underlying biology from different perspectives, increasing the chances for identifying effective biomarkers. Ideally, these perspectives could be integrated in a common data analytical framework, to enable a joint (or multimodal) data mining and decision [2].

Traditionally, the methods for analyzing pathology images focused on extracting quantitative measures for a set of predefined morphological parameters (e.g. counting, classifying and characterizing the nuclei) and on reproducing the expert’s decision in diagnostic applications (for a review see Gurcan et al. [3]). More recently, a number of applications of pathology image analysis combined image-based quantitative features with genomic information. For example, Yuan et al. [4] showed that nuclear morphometry is an independent prognostic factor that can improve a genomic signature. A similar approach is discussed by Kong et al. [5] in the case of glioblastoma where they show how nuclear and cytoplasmic features can be linked to genomic profiles and survival outcome. More advanced techniques combine several image-derived characteristics, such as co-localization of tumor nuclei and lymphocyte infiltration [6]. In all these cases however, the imaging features were predefined and based on previous known associations between histopathology and diagnostic/prognostic.

Our interest is in developing a more general computational framework that would allow the integration of the standard histopathology images in the biomarker discovery workflow and in which the image features would be learned in a data-driven fashion, enabling a prior-free data mining. The main challenge when analyzing the pathology images stems from their high complexity and size, and seeming incompatibility with *omics data. In the present work we propose to use the *bag-of-features* approach [7] for reducing the dimensionality of the images and extracting salient features. This approach has already been used in histopathology image classification applications [8, 9] and has the main advantage of allowing an unsupervised learning of image representation. The features extracted describe mostly the textural appearance of small neighborhoods and may be combined with other types of features (e.g. nuclear morphometry) in later stages of image analysis, but these approaches will not be discussed here. As an alternative to bag-of-features, one could use deep learning methods for learning image features as proposed by Cireşan et al. [10] or Cruz-Roa et al. [11]. However, these methods require a larger sample size and were applied in a supervised learning context.

We propose a novel representation of histopathology images which extends the standard bag-of-features with a number of derived measurements aimed at capturing more global characteristics of the tissue sample. In addition, we introduce an objective criterion for optimizing the image representation. The new computational framework is demonstrated in a biomarker discovery scenario, where prognostic features (both imaging and gene expression) for relapse-free survival in breast cancer are sought. We see the application of this approach as a succession of two independent steps, not necessarily performed on the same data corpus. In the first step, a histopathology image representation is learned from a collection of images representative for the pathology under investigation. In the second step, the images of interest are recoded based on the constructed representation and the resulting image features are jointly analyzed with the molecular and clinical data.

## Methods

### Data

The data used in this study is a subset of the data from Moor et al. [12], selected solely based on the availability of the material for analysis. Overall there were *n*=196 standard pathology (haematoxylin-eosin-stained) slides with breast tissue sections, not all containing a tumoral component and not necessarily from different cases. All images were obtained by whole-slide scanning of the pathology slides at 40× magnification, resulting in color images of about 150,000×100,000 pixels.

These data were partitioned into an image model learning set (*n*=131) and a biomarker discovery/data mining set (*n*=65). In the biomarker discovery set we kept unique cases for which the slides contained >70 *%* tumor component and the clinical, survival and gene expression data were all available. The expression profiles of 47 target genes (including 5 control genes) were obtained by quantitative real-time PCR (qRT-PCR). A full description of the data set is available in Moor et al. [12] and the major characteristics of the biomarker discovery set used here are given in Additional file 1.

We computed the genomic prognostic signature (PRO_10) as described in Antonov et al. [13] for all the cases with full genomic profiles.

### Image processing

#### Preprocessing

All images were downscaled to an equivalent of 2.5× magnification by subsampling the Gaussian-filtered higher resolution images (the 4-th level in a Gaussian pyramid). In the resulting images a mask corresponding to the tissue regions was obtained by adaptive thresholding in the green channel. The mask was subsequently refined by morphological operations: erosion with a circular structuring element with radius 13 followed by gap filling and removal of small objects.

For each image we estimated the intensity of haematoxylin (H) staining by deconvolving the RGB-images as described by Ruifrok et al. [14]. The intensity levels of the haematoxylin image (H-image) were adjusted by adaptive histogram equalization. Finally, the background pixels were masked out using the tissue region mask computed as above. In all subsequent image processing steps, only the H-images were used.

#### Learning the image representation

The bag-of-features [7] approach has two main stages: (i) learning an appropriate *codebook* for representing the images of interest and (ii) re-coding the images based on the frequencies of each *codeblock* (codeword from the codebook). Thus, the resulting representation of the image is a histogram of the codeblocks. For the current application, we extended this representation to include several derived features. We point out that once an appropriate image representation is learned, it can be applied unchanged to other similar image collections thus this step does not need to be repeated on each new data set.

##### Codebook learning

The codebook is a collection of representative local descriptors $\{C_{1},\dots,C_{K}\}$ obtained as centers of *K* clusters resulting from *k*-means clustering of a number of image local descriptors (i.e. a vector quantization procedure). For this, the images are decomposed in a set of local neighborhoods for which descriptor vectors are computed. The local descriptors range from pixels intensities to responses to filter banks or other textural descriptor. For the histopathology images, the Gabor wavelets provide a good set of descriptors, so they were adopted in the present work. Each local neighborhood of size *w*×*w* was convolved with a bank of 24 Gabor filters [15],

$${} G(x,y; \nu, \theta, \sigma) = \exp\left(-\frac{x^{2}+y^{2}}{2\sigma^{2}} \right) \times\exp\left(2\pi\nu j(x \cos\theta + y \sin\theta) \right) $$

where $j = \sqrt {-1}, \nu $ was the frequency, *θ* the orientation and *σ* the bandwidth of the Gaussian kernel. These parameters were set to $\sigma \in \{1, 2\sqrt {2}\},\theta \in \{k\frac {\pi }{4}|k=0,\dots, 3\}$ and *ν*∈{3/4,3/8,3/16}, respectively. They were kept fixed throughout all the experiments. For each filter response, its mean and standard deviations were recorded, thus each local neighborhood *w*×*w* was represented by 48 values (24 means and 24 standard deviations). A comparison of Gabor wavelets with other local descriptors, in the context of histopathology image analysis, is given by Budinská et al. [9].

The size of the codebook (i.e. the number of clusters in *k*-means clustering), *K*, is a free parameter that has to be chosen/guessed at the moment of codebook construction [8]. It can also be optimized for the problem at hand [9] using, for example, the Gap statistic [16]. Here we took advantage of having available a number of examples for different tissue components (fat, fat foamy macrophages, comedo necrosis, connective tissue and carcinoma infiltrating fat – for examples see Additional file 1) which we used as reference categories. The goal was to choose the size of the dictionary *K* in such a way that the representations of these categories are sparse and have a minimal overlap. For each image *i*, let *y*_*i*_= {*j* | if codeblock *C*_*j*_ is used in coding the sample *i*}, be the set of codeblocks used in its coding. Then we define the following quantities (where |·| denotes the cardinality of a set):

- total Jaccard index,
  $$J(K) = 0.5 \sum\frac{|y_{i} \cap y_{j}|}{|y_{i} \cup y_{j}|}, $$
  where the sum is taken over all pairs (*i*,*j*) of images from different reference categories;
- total sum of within-cluster distances,
  $$D(K) = \sum_{k=1}^{K}\ \sum_{i\in\text{cluster}\ k}\|{\mathbf{x}}_{i} - C_{k}\|^{2}, $$
  where **x**_*i*_ are the descriptor vectors.

With these quantities, we defined an (empirical) objective function:

$${} \Psi(K) = \log\frac{n_{c}(n_{c}-1)}{2} - \log J(K)- \log\sqrt{D(K)} - 0.75\log K, $$

where *n*_*c*_ is the number of reference categories (in our case *n*_*c*_=5). The overall goal of our image recoding step is to find a low dimensional (sparse) representation which still bears enough information for discriminating major tissue components. For this, we minimize *J*(*K*), i.e. the overlap between the representations of the reference categories. At the same time, we require tight clusters (small within-cluster total distances *D*(*K*)) and sparse representation (small *K*). Hence, the desired value for *K* is the one that maximizes *Ψ*(*K*), where we note that the first term is constant (included to bring the values closer to 0) and that the scaling factor 0.75 is used to reduce the influence of *K*.

##### Image recoding

**Fig. 1:**
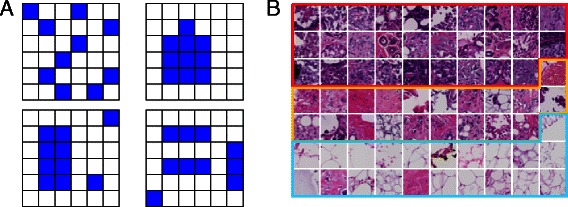
Codeblocks and codebook. **a** An example of four different hypothetical distributions of the codeblocks leading to identical frequencies. To cope with such situations, the distribution of codeblocks is also taken into account through extended image features. **b** A visual representation of the obtained codebook. The 70 image patches are the closest to the codeblocks obtained after *k*-means clustering. The three groups of codeblocks (with 29, 20 and 21 elements, respectively) correspond to the major clusters in Fig. 2 and the ordering of the image patches is the same as in the clustering

Once a suitable *K* is found and a codebook is constructed by *k*-means clustering, the standard bag-of-feature approach represents the images as codeblock histograms. However, in this coding, all spatial information about the distribution of the codeblocks is lost. Consider the situation in Fig. 1a: all four images have the same number of patches assigned to the same codeblock, but the spatial arrangement is very different. In order to characterize these spatial differences, we extend the image representation with a number of statistics on the distribution of the codeblocks. For a given image and for each codeblock $k\in \{1,\dots,K\},$ we construct a binary image in which 1s represent regions assigned to the codeblock and 0s everything else. In these binary images, the connected components (4-neighbor connectivity) define individual objects and for each of them we compute the area (in pixels) and the compactness index (ratio of the squared perimeter to the area of the object). Finally, for each image and each codeblock, we compute (i) the median area, (ii) the maximum area, (iii) the ratio of the maximum area to the total area of the objects, (iv) the skewness of the distribution of the area values and (v) the mean compactness. Thus, for each codeblock in an image, aside from its frequency, we add five new values aimed at characterizing the distribution of the codeblock in the image. We will refer to these additional quantities as the “extended set of features”. The final representation of an image has a length of 6*K*: *K* values for the codeblock histogram (the standard representation) and 5*K* values of the extended representation.

### Joint data mining

The new representation of the images allows for direct application of standard data mining techniques. In the case of multi-modality data mining, the choice of a proper similarity metric/measure is of crucial importance. Two main strategies may be attempted for defining a proper similarity: combination of single, modality-specific, metrics or building/learning a fully multi-modality metric. The first approach has the advantage of using established metrics usually resulting in easily interpretable models and facilitating the comparison with known results. The second approach promises to build a similarity metric that better exploits the multi-modality nature of the data. These ideas can be implemented, for example, in the context of kernel machines (such as Support Vector Machines) where composite kernels (based on closure properties – see [17] p.75) would represent a possible implementation of the first approach and multiple kernel learning [18] an implementation of the latter.

In the present work and in order to demonstrate the general analytical framework, we make use of standard statistical tools. We aim at identifying image features that could be linked to expression levels of the genes of interest (genotype-phenotype association) and potential image biomarkers that alone or in combination with gene expression can be used for defining a prognostic signature. Besides the gene expression, we also used a proliferation gene signature PRO_10 [12, 13], which was shown to be prognostic in various cohorts of patients with breast cancer.

To test the association between image features and tumor size (T) and grade (G) we dichotomized the clinical variables (T: {T1, T2} vs {T3, T4}, and G: {G1,G2} vs. G3, respectively) and used two-sided t-test, with 0.05 significance level. The association of image features with gene expression was assessed based on correlation test (Pearson) with significance level 0.05 and the condition that the correlation coefficient was at least 0.5 (in absolute value). We also used canonical correlation analysis (CCA) to study the associations between image features and molecular data with significance level of 0.05 for Wilks’ test. The association between image features and survival outcome (relapse-free survival – RFS) was tested using Cox proportional hazard models (log-likelihood test), with significance level of 0.05. The hazard ratios were estimated from interquartile range-standardized variables (both image and genomic variables). To test if an image feature improves the prognostic value of the gene signature, we tested the difference between the models with and without the variable of interest using likelihood ratio tests. To assess the difference in survival between two groups we used log-rank tests. We binarized the variables by their median value, into high- and low- expressions or values. Since the work reported here is purely exploratory and the sample size is rather small, no adjustment for multiple hypotheses testing was performed. We used hierarchical clustering (Ward method) with Euclidean distance between samples to cluster the codeblocks.

All statistical analyses were performed in R package for statistical computing (http://www.r-project.org) version 3.2.2.

## Results

### Codebook

The image analysis methods described above were implemented in a Python package (available at https://github.com/vladpopovici/WSItk), using the scikit-image [19] and Mahotas [20] libraries.

For the codebook construction we used only the modeling set of images, none of the image used in the data mining phase being used for learning the codebook. From each image, a set of 3000 random patches of size 32×32 was extracted and the corresponding Gabor descriptors computed (vectors of 48 elements). These descriptor vectors were clustered using the *k*-means algorithm to build the codebooks. We estimated the optimal (in the sense of the *Ψ* objective function, described above) codebook size by evaluating *Ψ*(*k*) for $k=10,20,\dots,1000.$ The optimal value was found to be *K*=70 (see Additional file 1 for a plot of *Ψ*(*k*)) leading to 420 feature vectors for each image. Since the codeblocks are centers of the clusters (the means of descriptor vectors assigned to the respective cluster), they might not necessarily correspond to observed image regions. Thus we selected the closest regions to the codeblocks (the corresponding descriptor vectors were the closest to the codeblocks) to provide an approximate visual representation of the codebook - Fig. 1b. In the following, to designate a specific codeblock from the codebook, we will use the notation *C.xy*. We have extensively investigated the stability of the learned codebooks and the resulting image representations and we found the process to be stable – see Additional file 1.

**Fig. 2:**
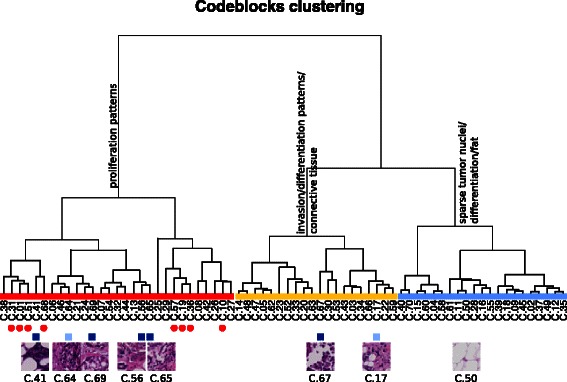
Hierarchical clustering of the codebook. Clustering the codeblocks led to identification of three major clusters, to which generic terms have been assigned. The codeblocks correlated with gene expression are marked with *red dots*. The codeblocks with potential prognostic value (in univariate analysis) are marked with blue squares (*dark blue* for *p*-value <0.01, *light blue* for 0.01≤*p*-value≤0.05

The hierarchical clustering of the codeblocks (Fig. 2) revealed a rather structured content: three major groups of codeblocks could be identified. We tentatively labeled them as “proliferation patterns”, “invasion/differentiation patterns/connective tissue” and “sparse tumor nuclei/differentiation/fat” to indicate the major components in the clusters - without claiming a precise histopathological characterization.

A number of codeblocks were found to be associated with tumor size (C.10, C.18, C.29, C.38, C.41, and C.42) and grade (C.09, C.34, C.43, C.45, C.48, and C.62).

### Correlations between image features and gene expression

The association analysis between image features and gene expression identified a number of significant (*p*<0.05 and *ρ*>0.5) pairwise correlations (all in the range 0.50−0.60). In all, eight different codeblocks were associated with different genes, most of them with *CCNE1* and *CCNB2*. The codeblock C.31 was associated with most genes (*CCNE1, CCNB2, BIRC5, PRC1, SPAG5*) either by its frequency of appearance in the image or by the skewness of its distribution. By summing the frequencies corresponding to image features that are highly correlated (e.g. C.38, C.31, C.01, C.51, C.41, C.68) the correlations coefficients were improved to 0.65−0.70. CCA confirmed the association between these image features and gene expression data (Wilks’ test *p*=0.026). The image features C.10, C.19, C.57, and C.68 and the genes *CCNE1*, *CCNB2*, and *SPAG5* had the strongest impact on the canonical dimensions. These were also the most stable image features-gene expression correlations in the image representation stability experiments – see Additional file 1.

Despite the fact that the PRO_10 gene signature is an average of proliferation genes which were found to be correlated with image features, the correlations between image features and PRO_10 did not reach the required significance level in all but one case: the skewness of codeblock C.31.

### Survival analyses

The goal of the analyses performed was to assess the utility of image-based variables for predicting relapse-free survival independently, or combined with the PRO_10 signature. In the set of samples analyzed, the genomic score is a strong prognostic marker (Cox regression: *p*=0.001,HR=2.12,95 *%* CI=(1.29,3.51)).

Univariate Cox proportional hazards models were fit for each of the 420 image features resulting in the identification of several significant associations with relapse-free survival endpoint. The most prognostic image features were C.41, C.56, C.65, C.67, C.69, with *p*<0.01 and HR between 1.16 and 1.70. From the extended set of features, the median area of the regions assigned to clusters C.15 and C.26 were significantly associated with RFS (*p*<0.05). The strongest predictor among the image features was C.69 (*p*=0.0018,HR=1.7,95 *%* CI=(1.22,2.37)).

In combined models (image feature and genomic score) a number of image features led to improved models (likelihood ratio test *p*<0.05), most of them from the extended set of features. From all these image features, C.69 remained significant in the multivariate model (with PRO_10) and had no significant interaction with the genomic signature.

**Fig. 3:**
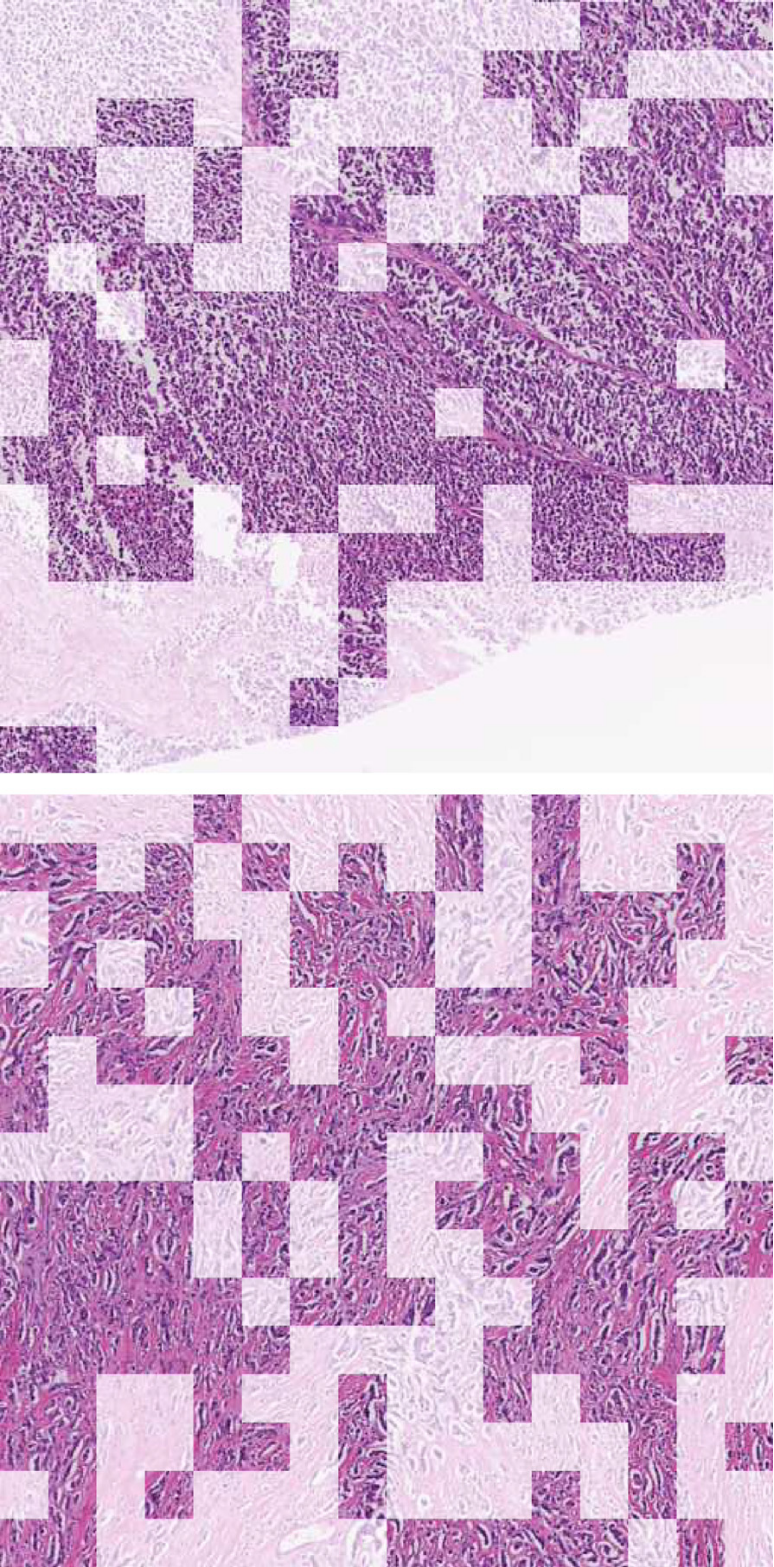
Regions assigned to the most prognostic codeblocks. 512×512 regions from two different samples with high image score (high risk of relapse), at 2.5× magnification. The image patches represented in full color were assigned to one of the C.41, C.56, C.65, C.67 or C.69 codeblocks. In Additional files 2 and 3, the corresponding whole slide images are provided

**Fig. 4:**
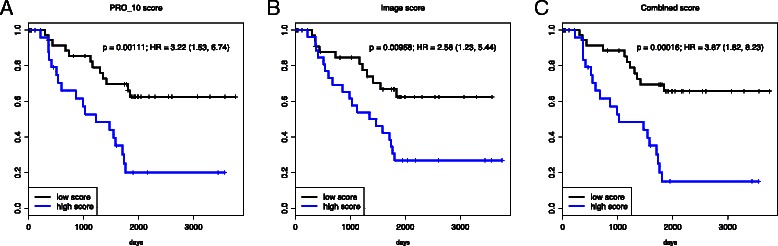
Kaplan-Meier curves for binarized scores. The genomic (**a**), image-based (**b**) and combined scores (**c**) were binarized by the respective median values into “low score” (low risk) and “high score” (high risk) categories. The combined score slightly improves on the genomic score

**Fig. 5:**
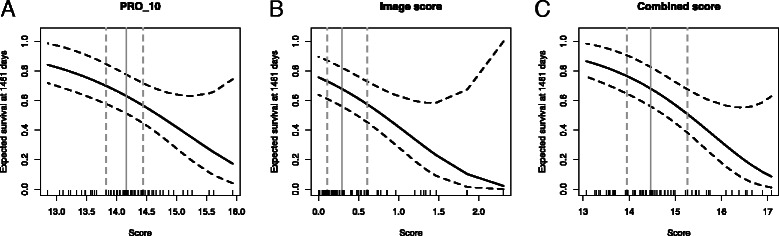
Prognostic scores at 4 years. Predicting the likelihood of an event (relapse) at 4 years, based on genomic signature (PRO_10 - panel **a**), the image-based score (panel **b**) and the combined score (panel **c**)

We defined an image score variable by averaging C.41, C.56, C.65, C.67, C.69 which resulted in a stronger prognostic factor (Cox regression: *p*=0.0003 and HR=1.76,95 *%* CI=(1.30,2.40) - see also Figure 3). In a regression model including the genomic and the image scores, both remained independent significant variables (PRO_10: *p*=0.05, image score: *p*=0.007, no significant interaction) and the model was signficantly better than the corresponding univariate models (*p*=0.013). In Fig. 4 the Kaplan-Meier curves for binarized (by median value) scores are shown, together with corresponding *p*-values (log-rank tests) and hazard ratios. Another visualization of the prognostic scores is given in Fig. 5 where the expected survival at 4 years is shown as a function of the genomic, image-based, and combined scores, respectively. Two examples of high risk cases, according to the image-based score, are given in Additional files 2 and 3.

## Discussion

The main challenge in introducing the histopathology images in the general data mining biomarker discovery framework stems from their high complexity and low level of information representation. Thus, while the images contain a huge amount of data (in the order of 10^10^ pixels) the extraction of information implies a considerable effort. Traditionally, this effort is performed by the expert pathologists or, more recently, by using quantitative methods for measuring a set of predefined morphological aspects to complement the pathology report. In this work, we took a third approach, in which the image data is reduced to a number of essential patterns (the codeblocks) whose frequency and spatial distribution in the image is used for data mining. The codeblocks are learned independent of any prior knowledge about the images, potentially enabling the discovery of new image features not necessarily assessed during the pathology review of the cases. The obvious drawback is the difficulty of interpreting some of the patterns and the possibility of having also artifacts in the model. The adopted representation of local neighborhoods in the image (responses to a bank of Gabor filters) encouraged the identification of codeblocks with distinctive textural appearance (Fig. 1). This local appearance may be later on combined with a nuclei detector and classifier (as in Yuan et al. [4]), for example, to obtain a more comprehensive characterization of the image.

By examining the similarities between codeblocks, we identified three major aspects of the images that are captured: proliferation, invasion/differentiation (within connective tissue) and isolated tumor nuclei (within regions predominantly with fat component) (Fig. 2). This result combined with the observation that the whole third cluster did not contribute to the prognostic models, suggests a possible refinement of the current method, in which these regions with high fat content are discarded in an initial preprocessing stage and a more detailed model is used to characterize the remaining regions.

We demonstrated the integration of the image features in a standard biomarker discovery scenario, in which both image-genes correlations (precursors to genotype-phenotype associations) as well as various survival prognostic models were tested. Since the main purpose of this exercise was to demonstrate the integration of image features with genomic information and the sample size was relatively modest, we did not adjust for multiple hypotheses testing and restricted ourselves to an exploratory analysis. Thus the associations found, while hypothesis-generating, have to be taken with caution and more validation is needed.

Most of the genes in the panel were related to proliferation processes, thus it is not surprising that the correlations with image features involved almost exclusively these genes. The strongest associations were found with *CCNE1* and *CCNB2*. Somehow surprising, no significant correlation was found with *MKI67* gene, a common marker (with Ki-67 specific staining) for proliferation.

A number of image features were found to be prognostic for RFS and we proposed a simple image-based prognostic score which averages five basic image features. The new score is strongly prognostic and is not correlated with the genomic score considered (PRO_10). When combining the two scores in a multivariable Cox regression, the two remained significant (with a marginal significance for the genomic score) and independent predictors (no significant interaction) leading to an improved model. Thus, the image-based score can be used either alone - as a first line predictor - or in combination with the genomic predictor. These results also demonstrate the complementarity of the two modalities - histopathology imaging and genomics - and suggest that refined predictors can be built by a combination thereof.

It must be noted that the sample size and the number of events did not allow for more variables in the regression models. Further analysis of the scores (either image-based or combined) in the context of usual clinical predictors (TNM-staging, hormonal status, etc.) is required before a definite conclusion about its clinical utility can be drawn. Nevertheless, the image-based score can already be used in applications like searching or indexing in histopathology image archives.

## Conclusions

We proposed a general framework for integrating the histopathology images in the routine genomic data analysis pipeline. The image features used are based on the responses of Gabor filters applied to single channel images. The approach can easily be extended to exploit the full color information and to include other types of features.

When applying our method to a data collection of breast cancer samples, we were able to identify a number of associations between image features and gene expression levels. More importantly, several prognostic image features were identified, some of them complementary to the genomic score. Thus, we could build an image-based and a combined survival score, improving on the performance of the genomic score. These results must be validated in larger data sets.

The code implementing the methods described is made freely available and continues to be under active development.

## Availability of data and materials

The source code for the image analysis methods described in the paper is available from the GitHub repository https://github.com/vladpopovici/WSItk.

The data used to demonstrate the methods described is not publicly available.

## Ethics approval and consent to participate

The data used to demonstrate the methods in this study has been graciously provided by the Department of Medical Oncology, Inselspital Bern, Switzerland. All patients gave a general consent for the use of their tissue samples in research.

## Additional files

Additional file 1 Codebook construction details [PDF file]. The codebook was optimized based on a objective function and a set of reference categories. This file contains the plot of the objective function and example images for the selected categories. (PDF 12390 kb)

Additional file 2 High risk carcinoma according to image-based score (Example 1). [JPG file]. Whole-slide image of a tumor labeled as high risk by the image score, with the regions used in scoring highlighted. (JPG 9758 kb)

Additional file 3 High risk carcinoma according to image-based score (Example 2). [JPG file]. Whole-slide image of a tumor labeled as high risk by the image score, with the regions used in scoring highlighted. (JPG 12800 kb)

## Abbreviations

HR: hazard ratio
qRT-PCR: quantitative real-time polymerase chain reaction
RFS: relapse-free survival
