# Supplementary material for: Joint analysis of histopathology image features and gene expression in breast cancer
Source: BMC Bioinformatics. 2016 May 11;17:209. doi: 10.1186/s12859-016-1072-z (PMC4864935; doi:10.1186/s12859-016-1072-z)
Supplement: Additional file 1 — Codebook construction details [PDF file]. The codebook was optimized based on a objective function and a set of reference categories. This file contains the plot of the objective function and example images for the selected categories. (PDF 12390 kb) [file 12859_2016_1072_MOESM1_ESM.pdf]

Additional File 1: Codebook construction details.

1. Codebook optimization criterion

The objective function (codebook score) has been estimated for k=10, 20,…, 1000, and the optimal value was found to be k=70. The resulting values are shown below:

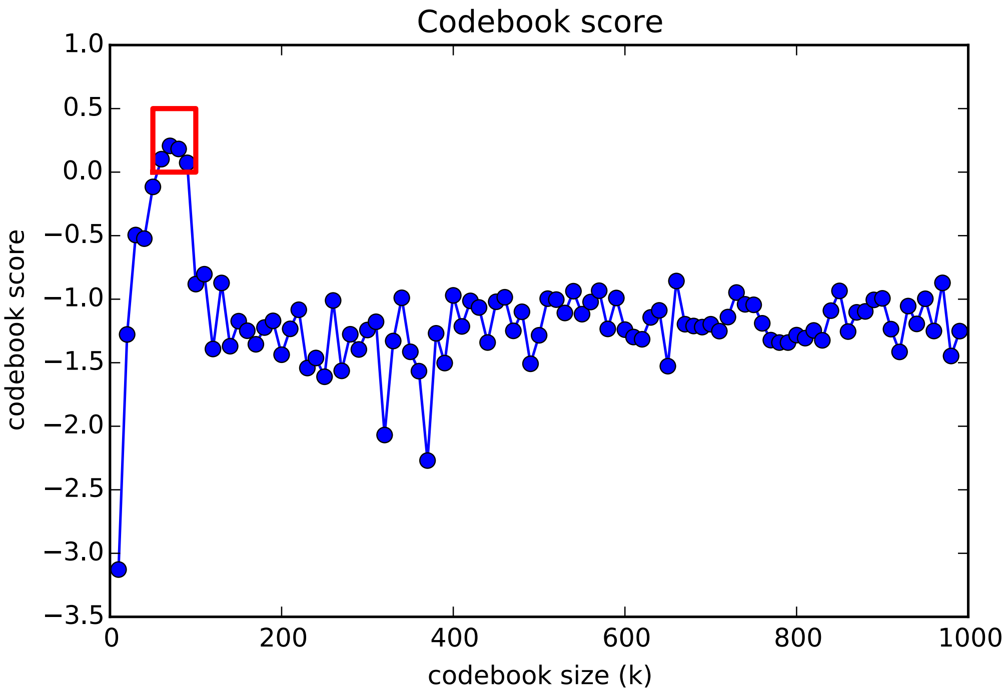

2. Reference categories for codebook optimization

Examples of images in the reference categories. The images have been cropped from slide images at 40x magnification (512x512 pixels).

2.1 Fat

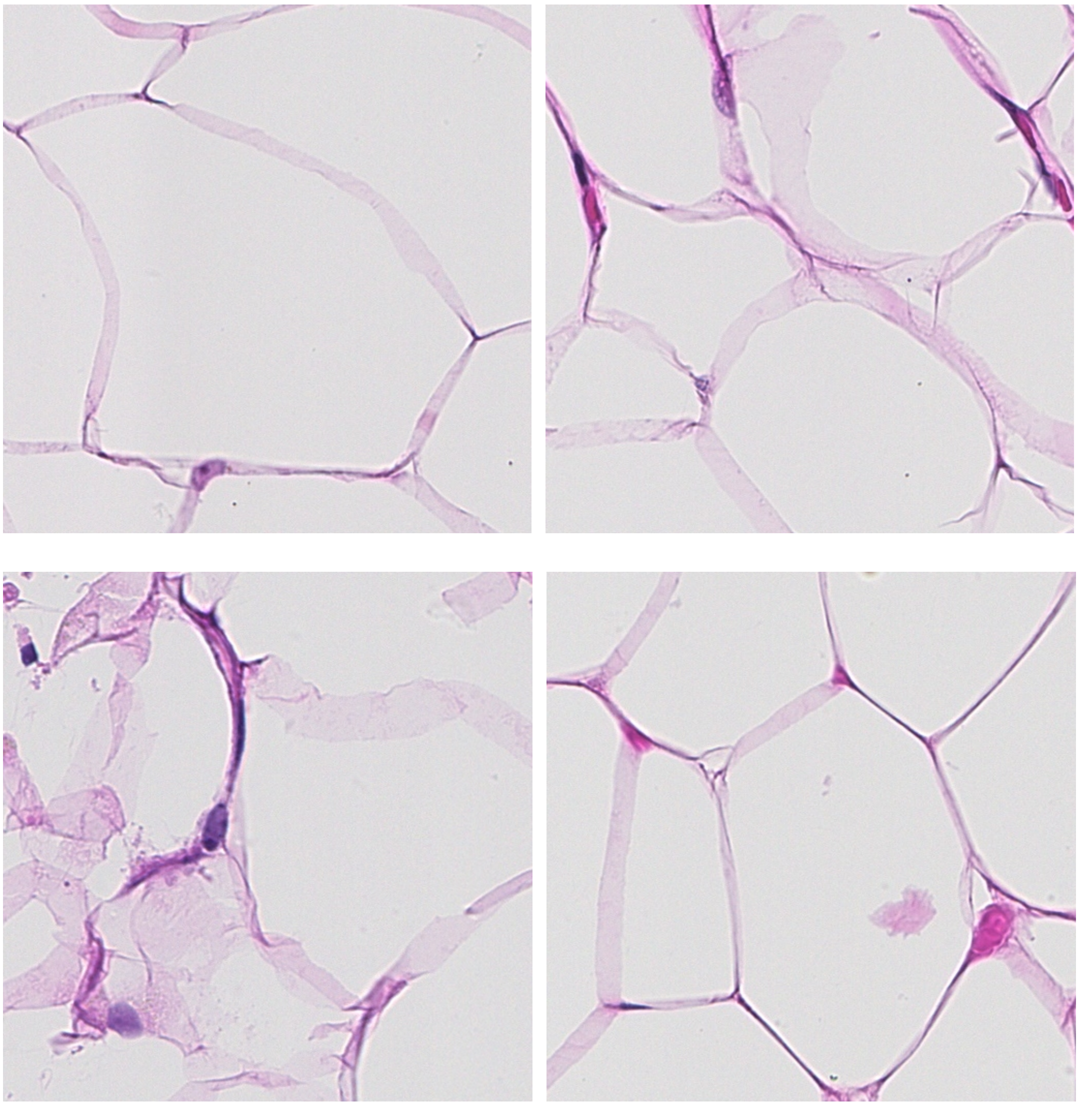

2.2 Fat foamy macrophages

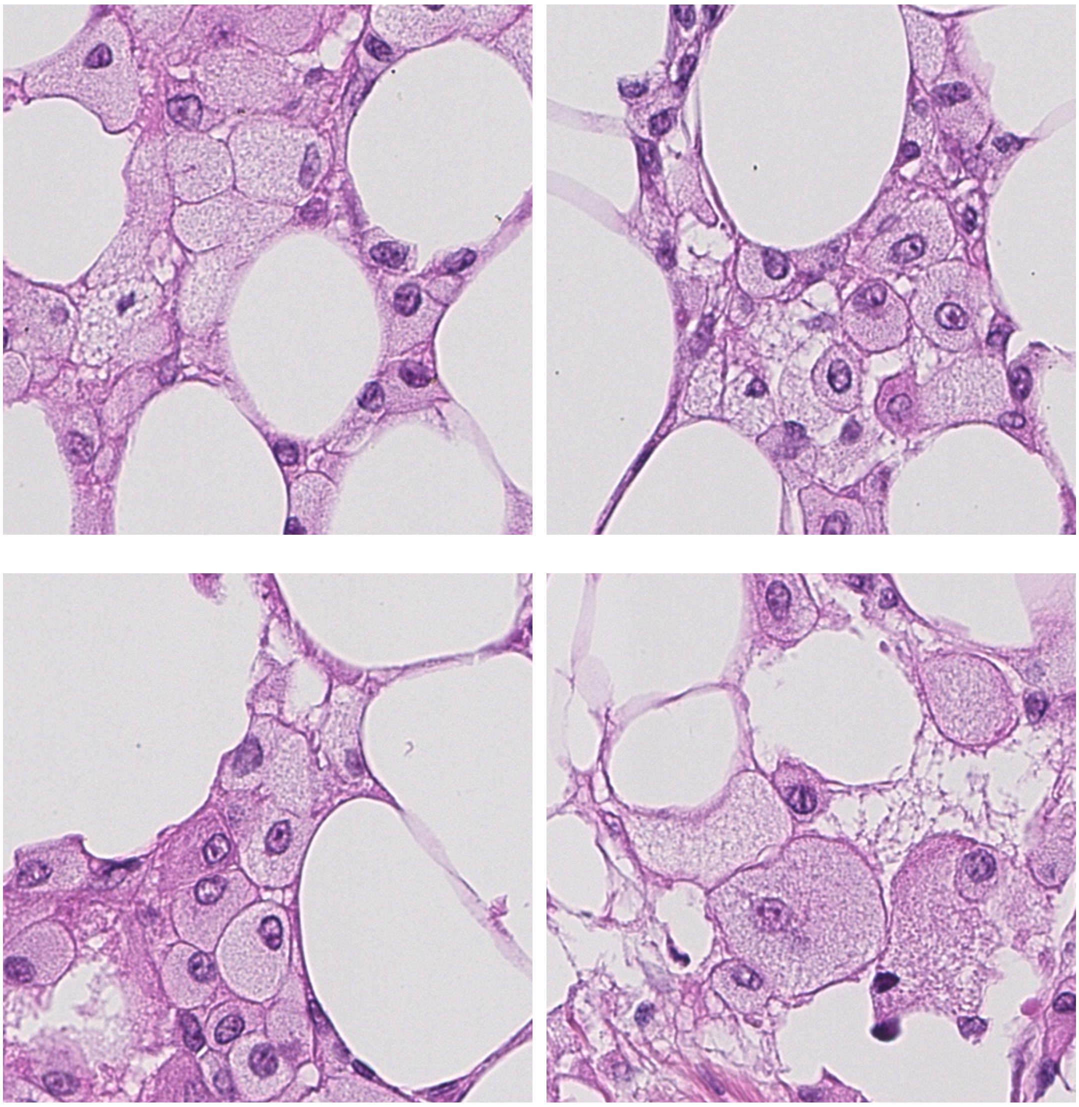

2.3. Comedo necrosis

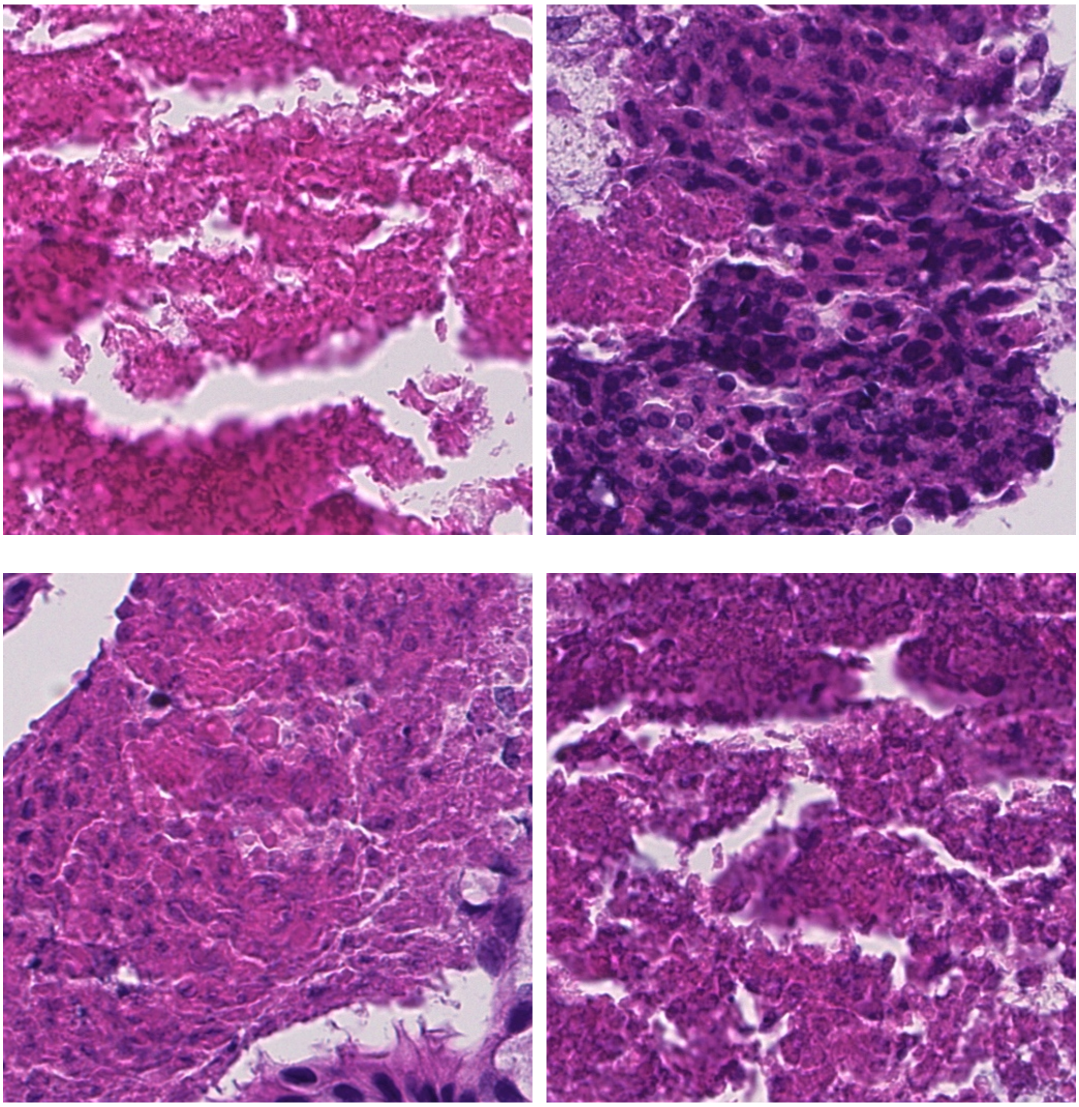

2.4 Connective tissue

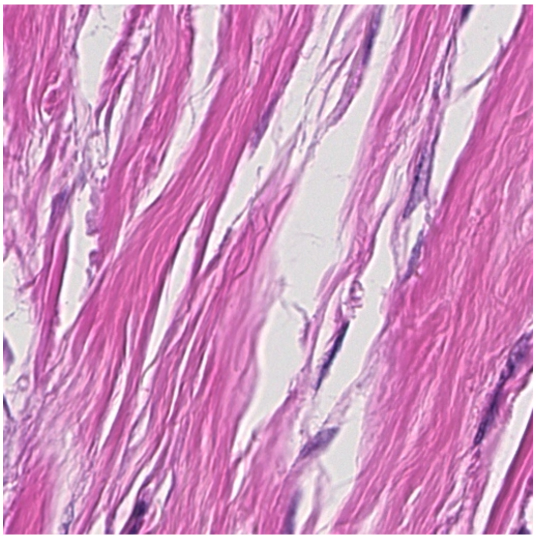
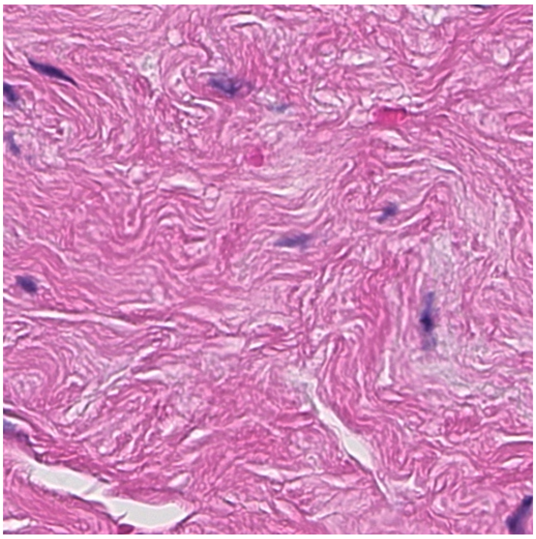
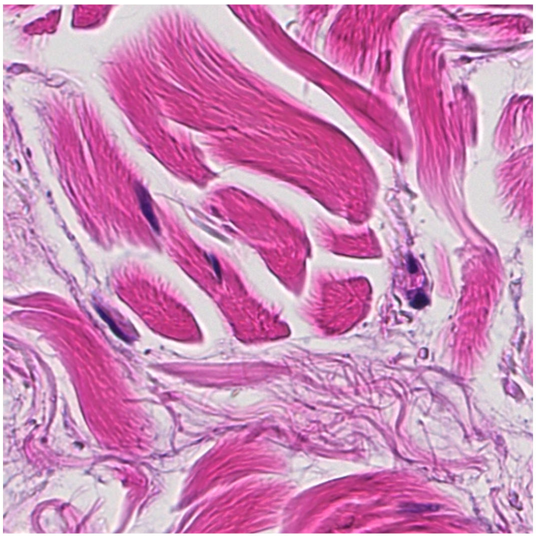
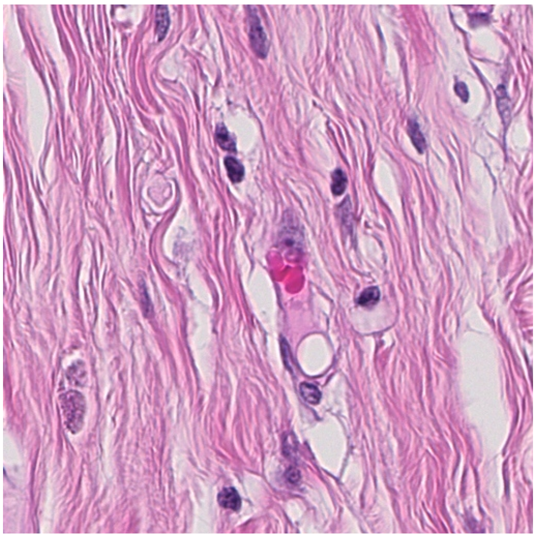

2.5 Carcinoma infiltrating fat

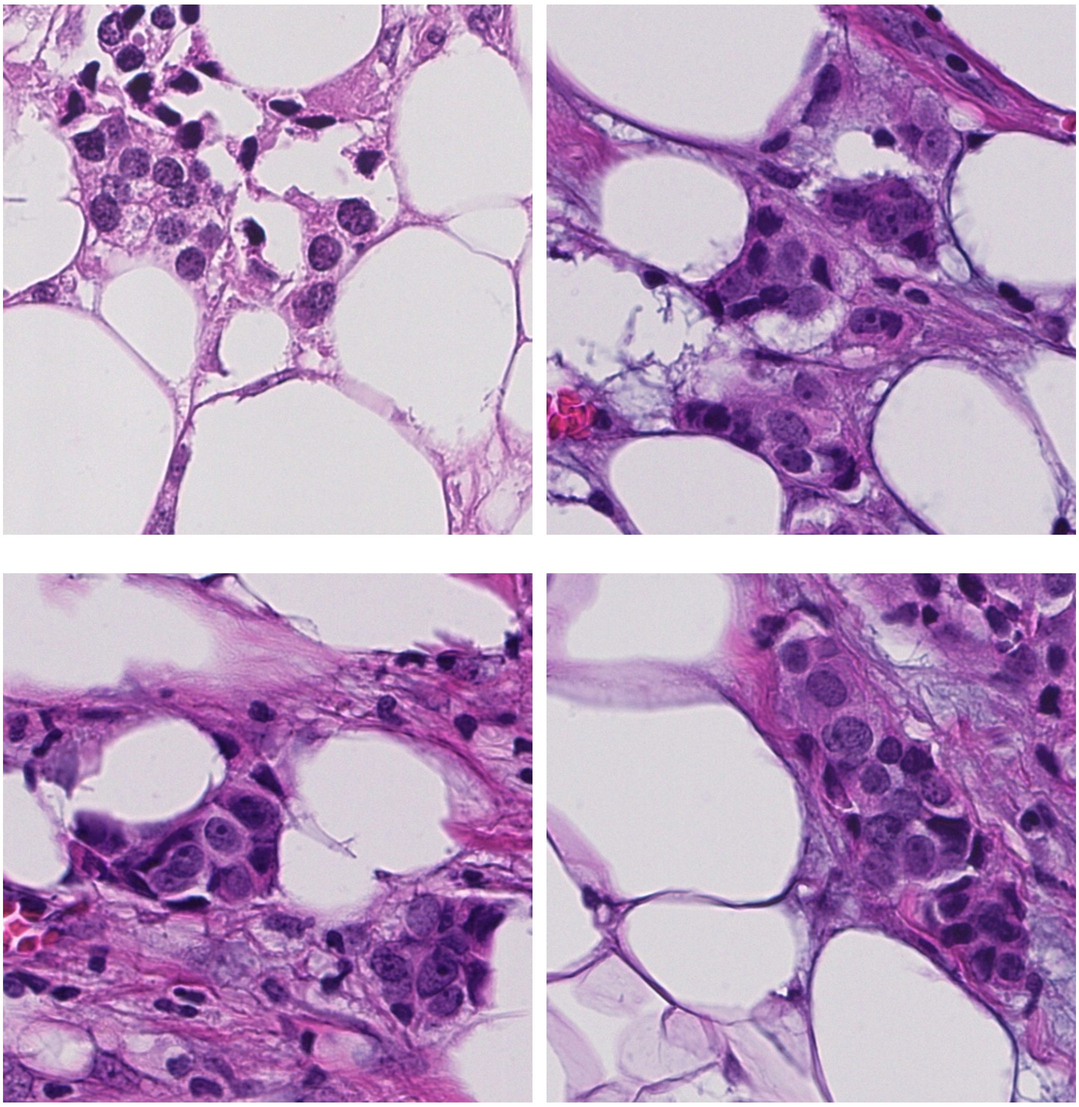

3. Stability of the codebooks

In order to investigate the stability of the image recoding phase, with respect to the change in the modeling set, we performed a number of simulations in which we repeatedly built codebooks based on randomly selected images and compared the coding obtained.

3.1. K-Means cluster centers stability

We simulated the construction of codebooks with increasing number of training samples (increments of 10 images, starting from 20 images) and we measured the distance (Euclidean distance) between cluster centers of consecutive codebooks. The following plot shows how the amplitude of the updates greatly reduces in the later steps:

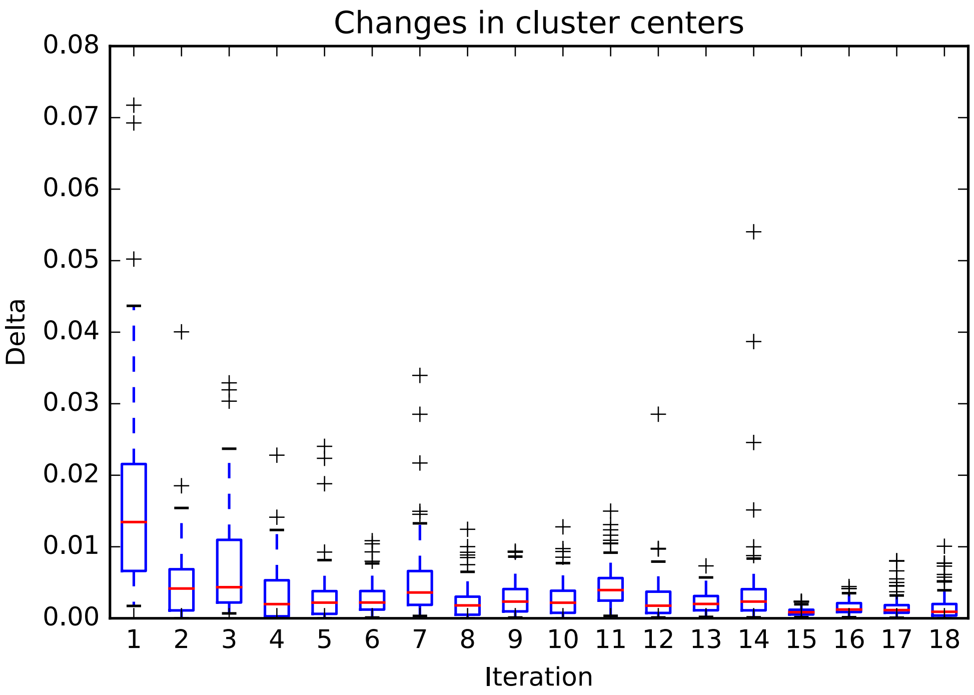

3.2 Image representation stability

To study the stability of the image representation, we generated a number of codebooks based on random subsamples of images, we aligned the representations and we measure the distances between random pairs of codebooks. In this study we used the set of 131 images (learning set). The simulations can be described as follows:

Initialize:

-number of simulations NS=100;
-number of randomly selected images in each simulation round NI=100;
-number of randomly selected patches from each image NP=3000;
-codebook size K=70

Simulations:
For s = 1,…, NS
-Select NI images from which draw NP patches
-Build the codebook C(s) and recode the images with this codebook

Repeat 1000 times:
-Draw randomly a pair (I,j) (1<= I,j <=NS) such that i != j
-Align the codebook C(i) with codebook C(j): since each codebook is the result of a k-means clustering, there is no guarantee that the k-th codeblock in C(i) corresponds to the k-th codeblock in C(j). For this we find a permutation P such that P(C(i)) will minimize the distances between corresponding codeblocks.
-Apply the permutation P to all images coded with C(i).
-For each image measure the distance between its representations under C(i) and C(j). Since these representations are histograms, the distance used is chi-square. Also, measure the distance between the representations under random permutations.

To assess the stability of the coding, we compared the distances between image representations under aligned codebooks and under random codebooks.

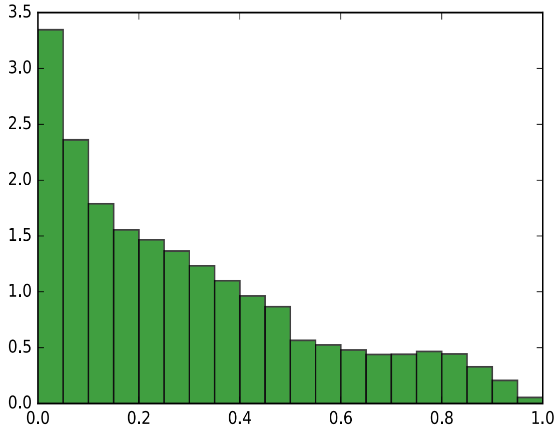

Figure 1 Distribution of Chi-square distances between pairs of codings.

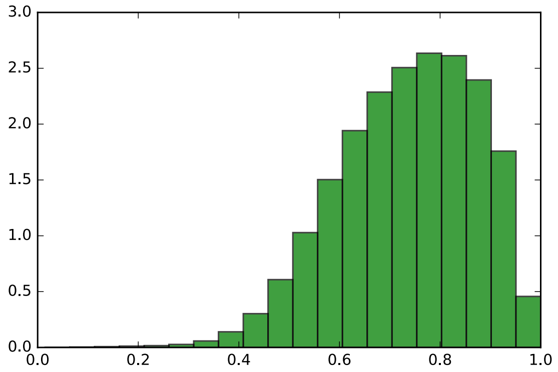

Figure 2 Distribution of Chi-square distances between random (non-aligned) codings.

3.3 Stability of the discovered correlations

Finally, we investigated the stability of the found associations under different codings of the images. For this, we generated 100 different codebooks and searched for correlations between image features and genes (see Methods section). The following plot shows for each gene the number of times it was found to be associated with at least one image feature:

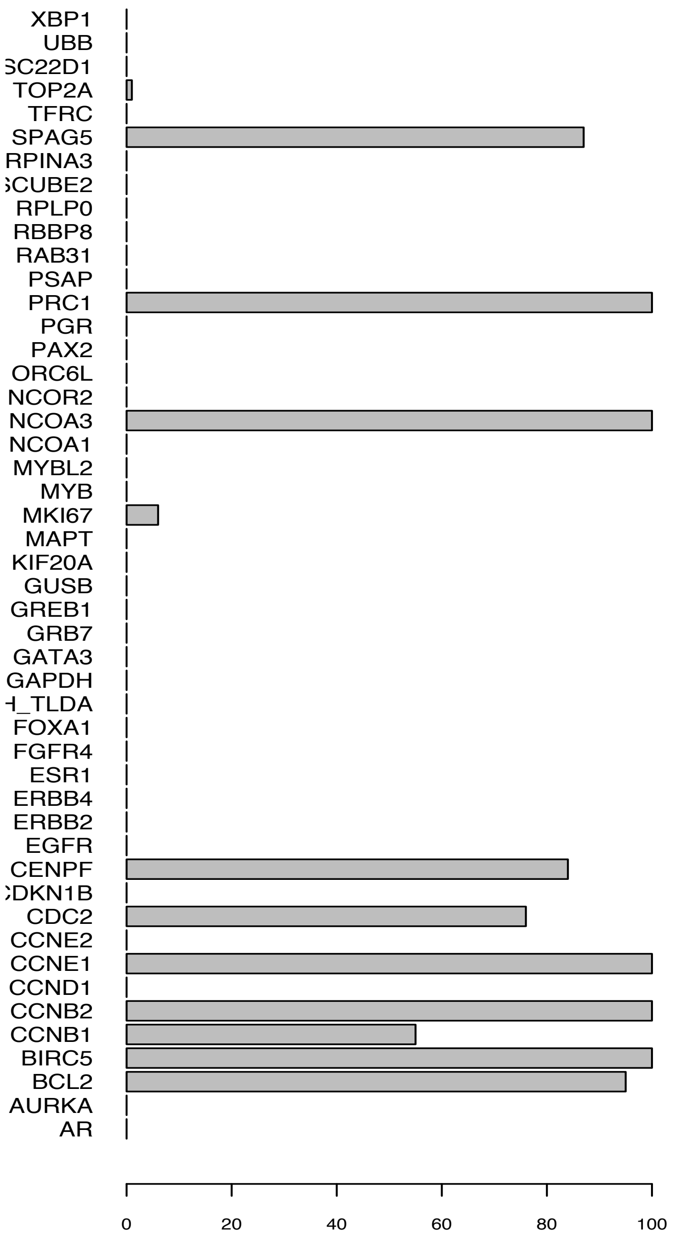

3.4 Leave-one-out validation

In order to estimate the validity of the associations between image features, on one hand, and gene expression levels and survival data, on the other hand, we performed a leave-one-out experiment in which 131 different codebooks have been constructed from the model learning set with each image excluded once from modeling. We note that, in general, when using cross-validation (in any of its forms) one has to ensure that the training set is representative for the population which, for histopathology images, translates into ensuring that all the histological and morphological aspects deemed important are present in the training set. Using leave-one-out ensures this aspect in the case of our modeling set.

To make the results comparable, the clusters were re-labeled such that the labels from the final model and those in the leave-one-out models represent the same concept (image appearance). This procedure involved finding, for each cluster center in leave-one-out codebooks, the closest cluster center in the final model, with ties assigned randomly.

The images from the biomarker discovery set were recoded with each of these 131 codebooks and the associations between image features and clinical and molecular data mined.

A first observation is that the codebooks are very stable, the distances between centers of each leave-one-out codebook and the final codebook being very low (compare with 3.1 above):

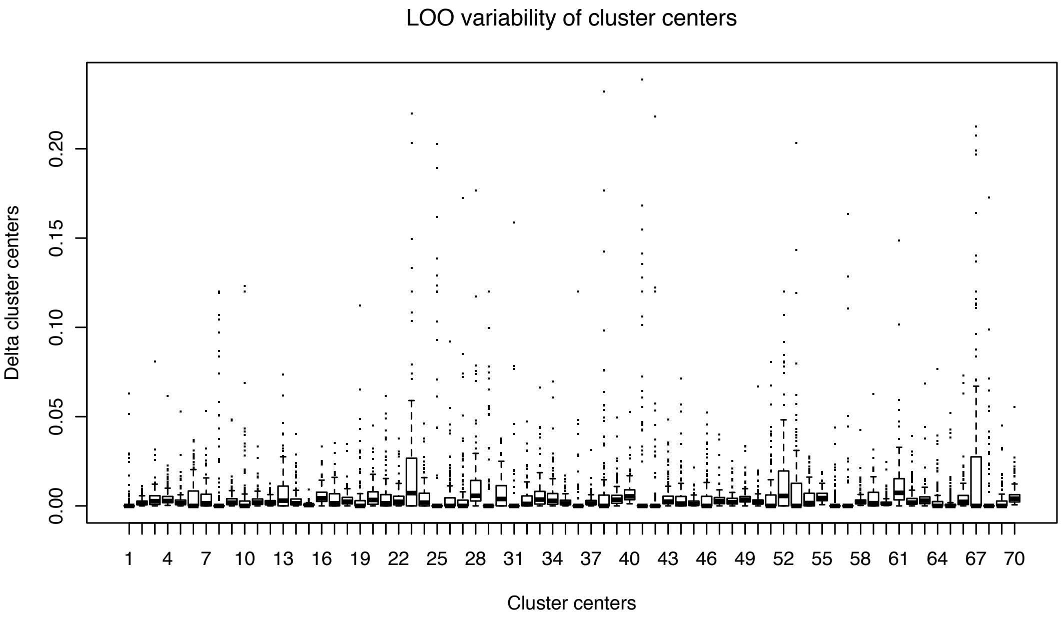

As a consequence, all the associations between frequencies of image features (codeblocks) and gene expression data, were re-found in each of the 131 iterations, with some new, sporadic ones, appearing as well:

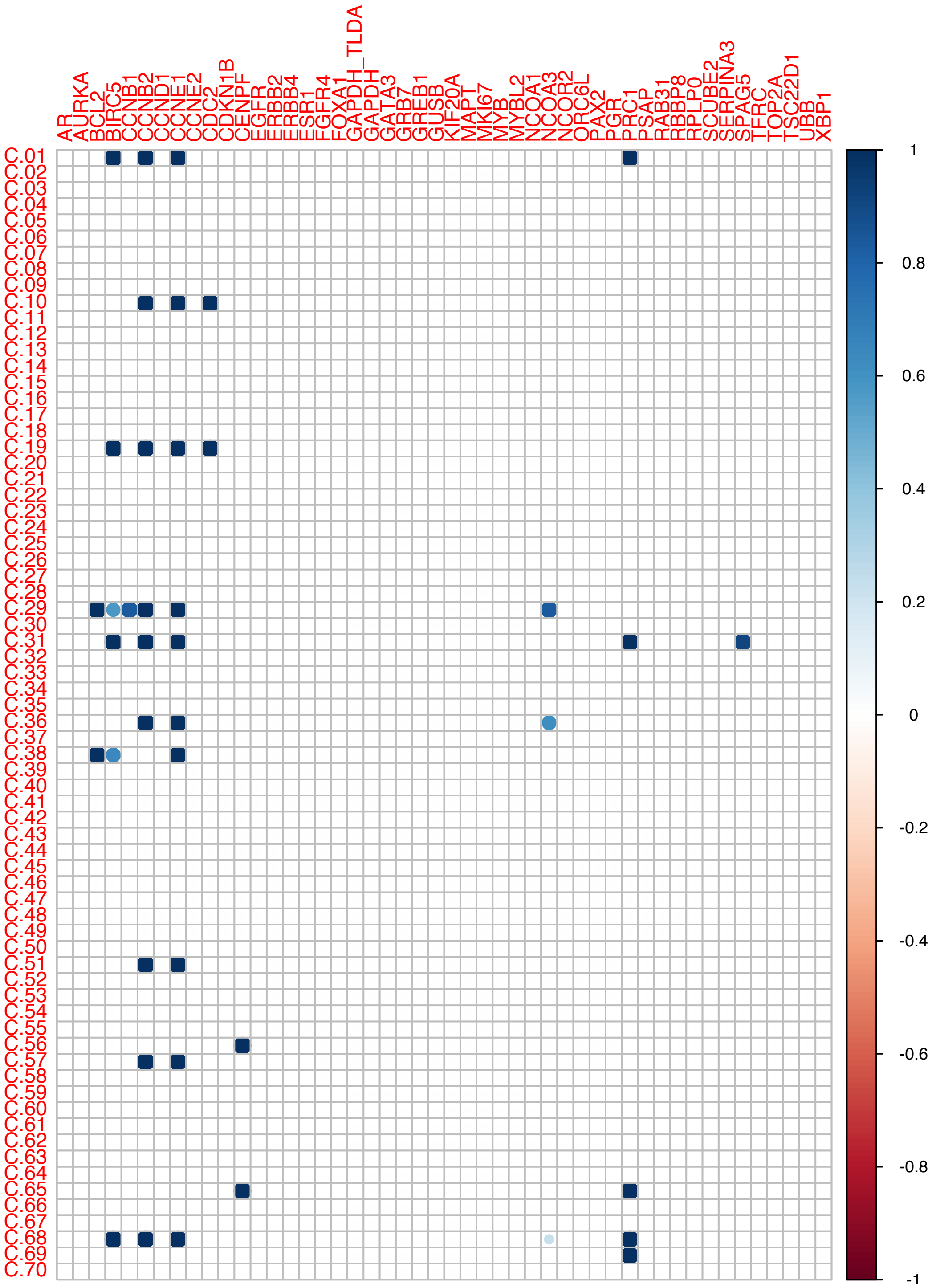

For each pair (image feature – gene), the figure shows the proportion of times the pair led to a significant correlation in leave-one-out experiments.

We also tested the association with survival data, and found C.41, C.56, C.65, C.67 and C.69 to be significant predictors for relapse-free survival in all the 131 cases of leave-one-out models.

4. Biomarker discovery set characteristics

Characteristic	Counts
**Menopausal category**	
**Postmenopausal**	65
**Premenopausal**	0
**Tumor size**	
**<= 2cm**	27
**> 2cm**	38
**Tumor grade**	
**Grade 1**	4
**Grade 2**	43
**Grade 3**	18
**Nodal status**	
**Negative**	27
**Positive (1-3)**	25
**Positive (>3)**	13
**ER and PR status**	
**ER+, PR+**	55
**ER+, PR-**	8
**ER-, PR+**	2
**ER-, PR-**	0
**Her2 status**	
**Her2-**	25
**Her2+**	16
**Unknown**	24
