# Supplementary figures and images for: Joint analysis of histopathology image features and gene expression in breast cancer
Source: BMC Bioinformatics. 2016 May 11;17:209. doi: 10.1186/s12859-016-1072-z (PMC4864935; doi:10.1186/s12859-016-1072-z)

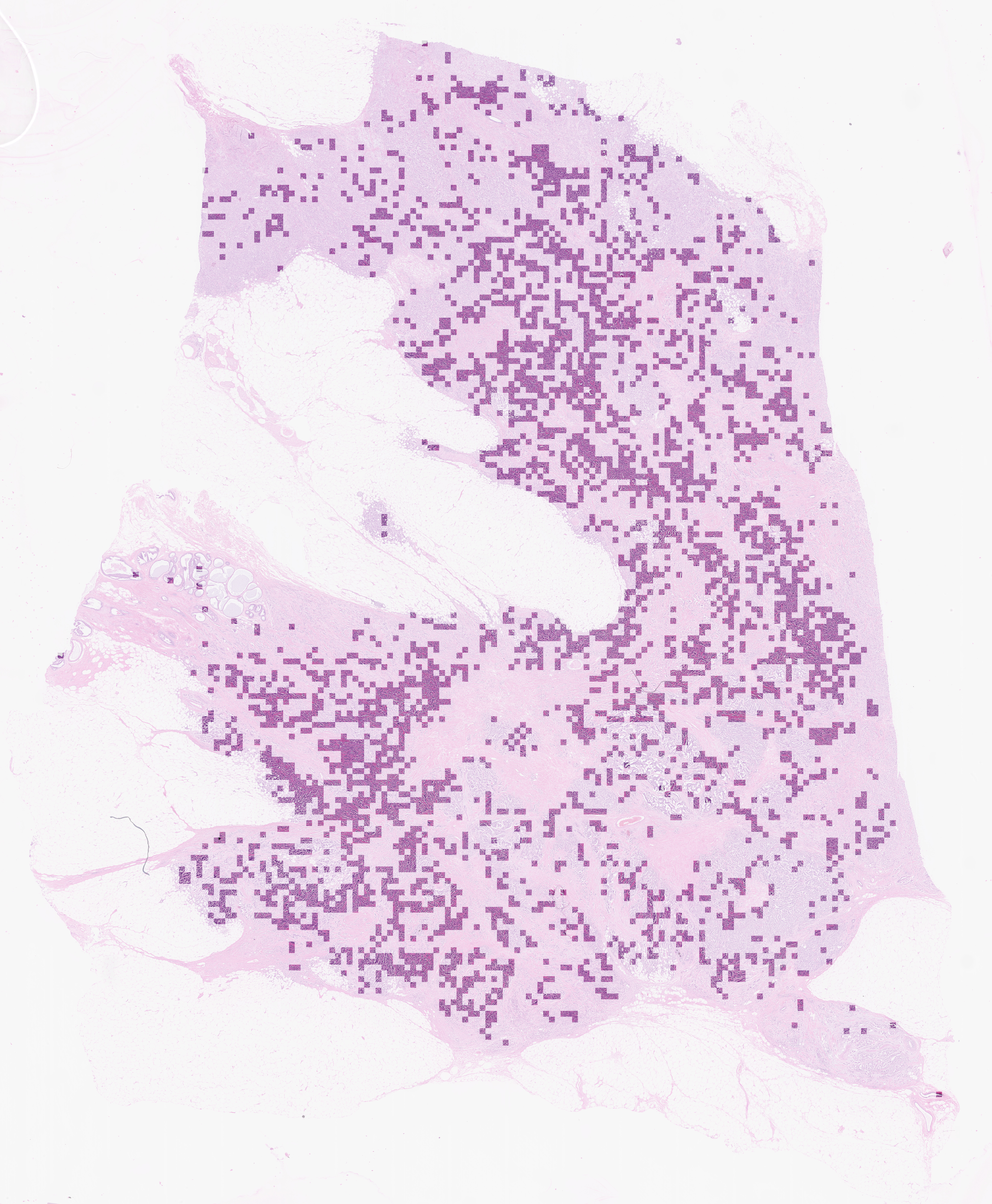

Supplement: Additional file 2 — High risk carcinoma according to image-based score (Example 1). [JPG file]. Whole-slide image of a tumor labeled as high risk by the image score, with the regions used in scoring highlighted. (JPG 9758 kb) [file 12859_2016_1072_MOESM2_ESM.jpg]

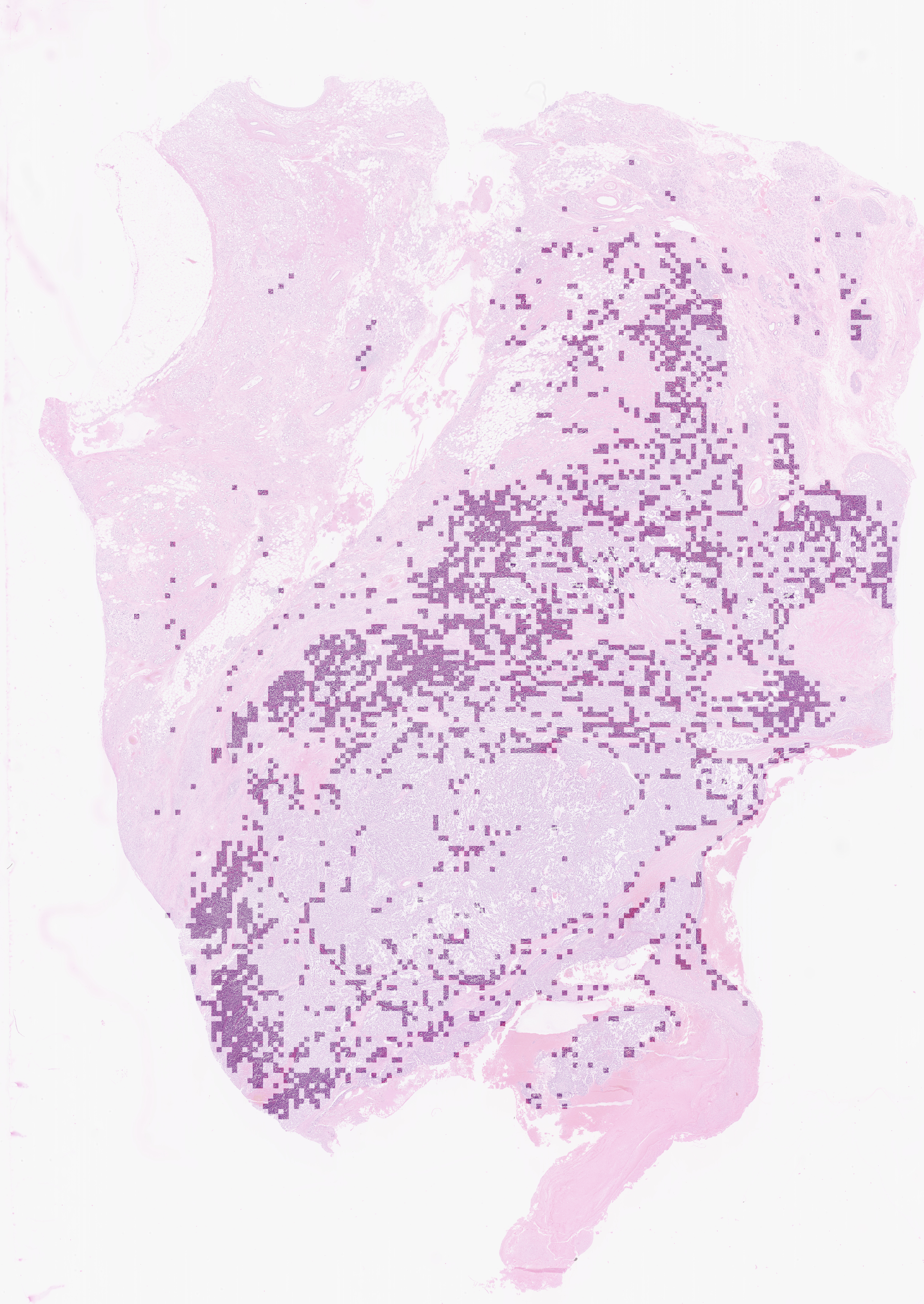

Supplement: Additional file 3 — High risk carcinoma according to image-based score (Example 2). [JPG file]. Whole-slide image of a tumor labeled as high risk by the image score, with the regions used in scoring highlighted. (JPG 12800 kb) [file 12859_2016_1072_MOESM3_ESM.jpg]
